# COMPLEX PANCREATIC RESSECTIONS WITH VASCULAR INVOLVEMENT IN JEHOVAH'S WITNESSES: HOW TO APPROACH?

**DOI:** 10.1590/0102-6720201600040019

**Published:** 2016

**Authors:** Eduardo de Souza Martins FERNANDES, Stéfano do Amaral FIÚZA, Felipe Pedreira Tavares de MELLO, Leandro Moreira Savattone PIMENTEL, Antonio Augusto Peixoto de SOUZA, Ronaldo de Oliveira ANDRADE

**Affiliations:** 1 Department of Surgery, Faculty of Medicine, Federal University of Rio de Janeiro; 2 Department of Liver Transplantation, Silvestre Adventist Hospital), Rio de Janeiro, RJ, Brazil

**Keywords:** Pancreatic neoplasms, Jehovah's Witnesses

## INTRODUCTION

Pancreatic cancer surgery is a technical challenge to the modern abdominal surgeon. Vascular resections, especially venous resections, have become frequent in specialized centers, accounting for 15-70% of duodenopancreatectomies and Appleby procedures for adenocarcinoma^11^.

In Brazil, pancreatic cancer represents the seventh cause of death due to neoplasm in men and the sixth in women^9^.

The religious group of Jehovah's Witnesses (JW) includes more than 1.3 million followers in Brazil, being the third largest JW community in the world, after the United States and Mexico, representing approximately 0.5% of the Brazilian population^2^.

The medical class faces ethical and legal challenges in the treatment of these patients, since this group refuses to receive blood transfusion or its components, often meeting with a medical duty to save lives. This ethical and legal dilemma causes many medical teams to refuse to perform surgical procedures in JW patients^4^.

Large operations, such as cardiovascular, thoracic, abdominal and organ transplants, have been performed in JW in a timid manner, with few citations in the literature. The principle of bloodless surgery, originally designed to rationalize the use of blood transfusions, was subsequently successfully applied to this population group, thanks to the advent of faster, more precise sealants, haemostatic agents and surgical techniques^4^.

## CASES REPORT

### CASE 1

A 69-year-old woman with a previous history of breast cancer and deep venous thrombosis and continuous use of rivaroxaban, presented with a weight loss of 12 kg in six months (normal weight of 84 kg) and jaundice, with metallic prosthesis internal biliary drainage. Computerized angiotomography showed a lesion in the head of the pancreas with involvement of the superior mesenteric vein, without lymph node involvement. She was submitted to four cycles of neoadjuvant chemotherapy (Folfirinox) and later referred for surgical treatment.

Surgical exploration did not reveal peritoneal carcinomatosis or hepatic metastasis. Duodenopancreatectomy was performed in conjunction with partial gastrectomy, cholecystectomy and extended lymphadenectomy with the use of a sealing forceps (LigaSure(r) 5 mm). After transection of the pancreas, a tumor adherence was evident from the right border of the superior mesenteric vein. Their partial resection was, then, performed with terminoterminal anastomosis without the need for grafting.

**FIGURE 1:**
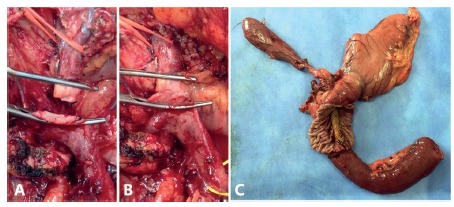
A) Resection of the superior mesenteric vein; B) its anastomosis in terminoterminal type; C) surgical specimen with exposure of the duodenum and metal prosthesis

Finally, reconstruction was performed in a single jejunal loop, with ductomucosa terminolateral pancreaticojejunal anastomosis with separate stitches, followed by a choledocojejunal biliodigestive anastomosis with continuous stitches, followed by a gastrointestinal anastomosis with separate stitches (oralis partialis) and an enteroentero anastomosis with continuous stitches (Braunn).

The patient received no blood products and the blood stove (CellSaver(r)) did not aspirate enough volume to be processed. Hemoglobin did not fall after the operation. Amylase dosage of drainage secretions on the 3^rd^ and 5^th^ postoperative days did not show pancreatic fistula. The histopathological findings showed invasive ductal adenocarcinoma of the pancreas with size of 4.5 cm and free surgical margins (R0). No lymphatic metastases was observed in the removed 31 lymph nodes.

### CASE 2

A 64-year-old woman with a history of Crohn's disease 18 years ago, and multiple episodes of acute cholangitis and pancreatitis with multiple hospital admissions for percutaneous drainage of peripancreatic collections, presented jaundice three months after initiation of treatment for tuberculosis. Image exams showed mass in the head of the pancreas and a prosthesis had been placed in the bile duct two months previously. Computerized angiotomography showed a lesion in the head of the pancreas with involvement of the portal vein, without lymph node invasion.

In surgical exploration, the pancreas presented diffuse enlarged consistency, with multiple adhesions and some peripancreatic collections, especially in the caudal region, closely adhered to the splenic flexure and left colon, without peritoneal carcinomatosis or evident hepatic metastases. Duodenopancreatectomy was performed in conjunction with partial gastrectomy, cholecystectomy, extended lymphadenectomy with the aid of a sealing forceps (LigaSure(r) 5 mm). After transection of the pancreas 3 cm from the pancreatic neck, tumoral infiltration of the portal vein was evidenced; partial resection of the vein and its reconstruction were performed, without the need for grafting. The surgical margin of the pancreatic border was positive for malignancy at the freezing test. Thus, total pancreatectomy with splenopancreatectomy was chosen due to the chronically diseased aspect of the remaining pancreas and, also, to the multiple previous pancreatic collections.

**FIGURE 2:**
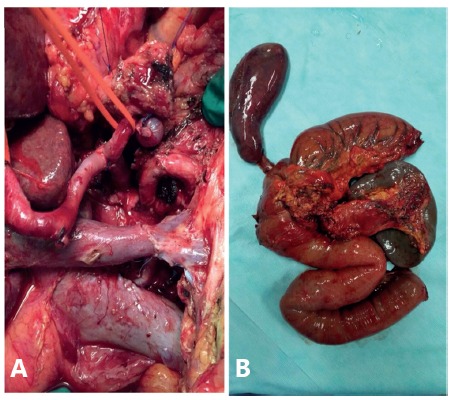
A) Portal vein terminoterminal resection and anastomosis; B) surgical specimen

Reconstruction consisted of a hepaticojejunal biliodigestive anastomosis with continuous suture, followed by a gastrointestinal anastomosis with separate stitches (oralis partialis) and an enteroentero anastomosis with continuous suture (Braunn). Abdominal drains were placed in a splenic store and close to the biliodigestive anastomosis.

The patient did not receive blood products and the blood stove (CellSaver(r)) recovered 192 ml of blood, which reinfusion was not indicated. Hemoglobin fell from 14.8 mg/dl to 10.7 mg/dl at the end of the operation. The patient received macronebulization with continuous infusion of vasoactive amines at low dose until the first postoperative day. Amylase dosage of drainage secretions on the 3^rd^and 5^th^ postoperative days did not show pancreatic fistula. Histopathological findings showed focal ductal adenocarcinoma of the head and neck of the pancreas, with extensive chronic associated pancreatitis, and free surgical margins (R0). Three lymphatic metastases were observed in the 32 lymph nodes removed.

## DISCUSSION

In the last two decades there has been considerable progress in the safety of pancreatic surgery, with important reductions in the rate of complications, need for blood transfusion and mortality. However, despite these improvements, it still presents great potential for bleeding, especially in cases of vascular resection, with great possibility of transfusion of blood products.

As a general rule, treatments not accepted by JWs are transfusion of whole blood, packed red blood cells, platelets, white blood cells, or plasma. Albumin, immunoglobulin, vaccines and clotting factors are of individual choice^3^.

The term bloodless surgery refers to a series of pre, peri and postoperative care measures aimed at reducing the need for allogeneic blood transfusion and improving outcomes. In the preoperative period the physiological reserve should be evaluated to estimate the capacity to withstand hypovolemia. The erythrocyte mass should be improved, even in the absence of anemia. Iron, vitamin B12, folate and erythropoietin may be required^4^ and are used in a variety of protocols.

The operation should be performed with meticulous hemostasis with electric or harmonic scalpel and hemostatics (bone wax, absorbable cellulose or collagens)^4^. Blood salvagers are capable of collecting and filtering blood drawn by aspiration or by compresses, which can then be reinfused into the patient^12^. Such use is already established in hepatic transplantation for hepatocellular carcinoma^1^ ^,^ ^5^. Some JWs may refuse the blood recovered from tampons since it has been interrupted in the blood circuit^12^. A meta-analysis of 10 studies in which blood recovery was performed showed no significant difference in the rate of recurrence of cancer among those who received recovered blood (n=741) and those who did not (n=1585). In some studies, the rate of cancer recurrence was even lower among those who used the equipment^5^ ^,^ ^12^, suggesting that there are deleterious effects of heterologous hemotransfusion on cellular immunity^5^.

Anesthetic staff may maintain controlled hypotension to minimize intraoperative bleeding. The technique is known as normovolemic hemodilution, which consists in removing part of the patient's blood intraoperatively, storing it and replacing the intravascular volume with crystalloids or colloids. Thus, blood lost during the operation will have reduced hemoglobin concentration and the removed blood will be available for transfusion (provided it is kept in a closed "blood circuit", as required by the JW). Breathing exercises and respiratory physiotherapy should be optimized to improve oxygen delivery. Hyperbaric oxygen therapy has been successfully used in some cases, with regimens of two to three times a day in patients with severe postoperative anemia, with hemoglobin levels up to 2.0 mg/dl^8^.
